# Feral Cats Are Better Killers in Open Habitats, Revealed by Animal-Borne Video

**DOI:** 10.1371/journal.pone.0133915

**Published:** 2015-08-19

**Authors:** Hugh McGregor, Sarah Legge, Menna E. Jones, Christopher N. Johnson

**Affiliations:** 1 Australian Wildlife Conservancy; Mornington Wildlife Sanctuary, PMB 925, Derby, Western Australia, 6728 Australia; 2 School of Biological Sciences, University of Tasmania, Private Bag 55, Hobart, Tasmania, 7001 Australia; University of Queensland, AUSTRALIA

## Abstract

One of the key gaps in understanding the impacts of predation by small mammalian predators on prey is how habitat structure affects the hunting success of small predators, such as feral cats. These effects are poorly understood due to the difficulty of observing actual hunting behaviours. We attached collar-mounted video cameras to feral cats living in a tropical savanna environment in northern Australia, and measured variation in hunting success among different microhabitats (open areas, dense grass and complex rocks). From 89 hours of footage, we recorded 101 hunting events, of which 32 were successful. Of these kills, 28% were not eaten. Hunting success was highly dependent on microhabitat structure surrounding prey, increasing from 17% in habitats with dense grass or complex rocks to 70% in open areas. This research shows that habitat structure has a profound influence on the impacts of small predators on their prey. This has broad implications for management of vegetation and disturbance processes (like fire and grazing) in areas where feral cats threaten native fauna. Maintaining complex vegetation cover can reduce predation rates of small prey species from feral cat predation.

## Introduction

The risk of predation on prey can vary across landscapes, ranging from areas of refuge where the impact of predation is low to areas with high predation risk and impact [1,2]. This variation is largely driven by differences in predator densities, by differences in spatial and temporal behaviours of predators, and by variation in hunting success by predators. Methods for measuring the first two drivers are well developed, however, measuring hunting rates and hunting success can be far more difficult. This is especially relevant for small terrestrial vertebrate predators, which are difficult to observe without altering their behaviour.

One of the key gaps in our understanding of the impacts of predation on prey is how vegetation structure affects the hunting success of small predators (‘mesopredators’). Many small predators require vegetative cover for concealment, to stalk and ambush prey [3,4], or for protection from larger predators [5]. Others depend on open vegetation to find and catch prey or allow vigilance for larger predators [6]. Whether open areas aid or inhibit predation is not broadly established for small predators.

Another key gap in our understanding is the role of surrounding cover in enabling stalks. A kill might occur in an open area, but the outcome determined by habitat provided on the approach [7]. This is known from larger predators such as lions [4], that require camouflaged stalks or ambushes in order to get close enough to initiate a successful hunt. Although numerous species of small predators have been observed to hunt in this manner, whether their success is quantitatively better has not been established. This is an important consideration, as it raises the question as to whether the impact of small predators would be greater in a purely open system, or in a matrix of open areas and cover.

Understanding such predation dynamics is important for conservation management, especially in grasslands and savannas. Within these ecosystems, many small animal species are threatened with extinction [8]. This is principally driven by recent intensification of disturbance regimes, such as grazing by domestic herbivores or increases in the frequency and severity of fire regimes [9]. These disturbances threaten small animals by either reducing food sources, or altering predator/prey dynamics. The latter is especially important in landscapes where introduced predators create inflated predation pressures [10], where cover from these predators is one of the few mechanisms allowing their survival. As low vegetation is one of the only forms of natural cover for small animals in these ecosystems, disturbance regimes like fires and intensive grazing that reduce vegetation cover could have profound consequences on these predator-prey interactions [11].

Here, we use animal-borne video-cameras [12,13] to document the hunting behaviour and success of completely wild feral cats *Felis catus*, in relation to vegetation structure in a savanna ecosystem. The feral cat is an invasive predator that threatens small vertebrates in many parts of the world [14–17], and is suspected of causing widespread declines of native mammals in our study area of northern Australia [18]. Circumstantial evidence suggests the impacts of cats are greatest where ground vegetation has been simplified by fire and grazing [19,20], and movement patterns of cats prefer areas with open and simple ground vegetation [21]. However, the link between the two has not yet been substantiated. We use our visual data to measure hunting success in different microhabitats, and test the hypothesis that the structural complexity of the habitat around the stalk and at the location of the kill affects hunting success. If complex microhabitats provide protection for prey, then removal of such would imply an increase in prey availability to cats. As most cats are likely ‘surplus killers’ [13,22], more food available would mean more prey is killed per night per cat. This would suggest that predatory impacts of cats could increase substantially if habitat is made more open, even if there is little change in prey densities, cat movements or cat densities.

## Materials and Methods

### Study area

The study was conducted on two large properties in the central Kimberley of north-western Australia (17°01’S, 126°01’E), one managed for commercial cattle production (Glenroy, 1500 km^2^) and the other a wildlife sanctuary managed by the Australian Wildlife Conservancy (Mornington Wildlife Sanctuary, 3200 km^2^). Vegetation is characterised by savanna woodland with a perennial grass layer, dissected by riparian vegetation along the edges of creeks. The climate is tropical monsoonal, with most rain (~750 mm/year) falling between December and April. All large introduced herbivores (cattle *Bos taurus*, horses *Equus caballus*, donkeys *Equus asinus*) have been removed from a 400 km^2^ fenced section of Mornington since 2005, with additional areas destocked in later years [23].

### Camera design and deployment

**Fig 1:**
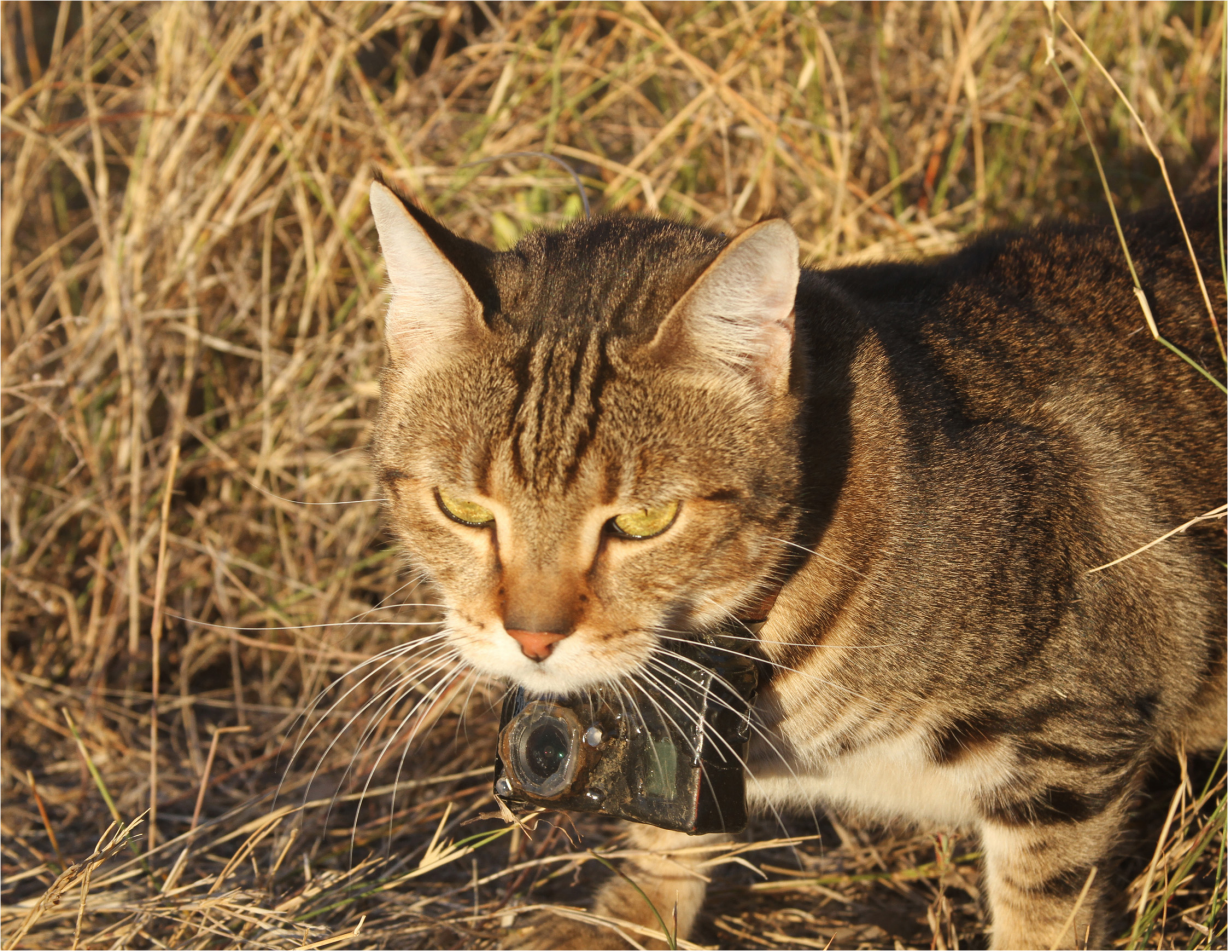
Photo of feral cat carrying the largest camera-collar used during this study (photo: Chelsea Parker).

We used two different types of video camera-collars, each with different methods of turning on to record video: manually or by remote UHF. All collars used a GoPro Hero 3 White camera (GoPro Inc, San Mateo, California, USA), with after-market modification. Cameras had their infra-red filter removed, and infra-red LEDs placed next to the lens (920 nm, with no visible light emission). For cats weighing more than 4.4 kg we attached one or two additional batteries (each 20 g, with 1300 mAh). All were packaged with a separate VHF beacon (Sirtrack, Havelock, New Zealand). Cameras turned on via remote UHF had a chip attached that triggered the camera with 433 mhz remote (Cam-do.com), which added an extra 8g to the camera. All components were packaged together on a collar, and outer-coated in two thin layers of epoxy resin. The finished camera-collars weighed 100−140 g (see Fig 1), with payload dimensions of 65 mm × 43 mm × 35 mm (+ 5mm for each extra battery). All packages weighted between 2% and 3.6% of cat body weight. Collar units were able to record 2 to 8 hours video.

Camera-collars were deployed on cats that had previously been captured for a study on habitat selection [21] and had been carrying a GPS collar weighing 110 g for at least a month, and were therefore accustomed to wearing a collar of this weight. Cats were re-captured with the assistance of muzzled dogs trained to locate and drive them up a tree. Once treed, cats were sedated with a dart containing the sedative tiletamine-zolazepam at a rate of 0.5 cc / kg shot from a Pnue-Dart X-calibre C0_2_ dart-rifle, then caught on a sheet stretched between two people as they fell from the tree. The GPS collar was removed, the camera-collar attached, and the cat released at point of capture once the sedative had worn off (4−6 hours later). In 16 deployments between 2012 and 2014, cameras were turned on just before release. To account for the possibility that behaviour immediately after release was abnormal, on a further seven deployments we remotely activated the cameras after at least 24 hours post release. In such instances, we either approached quietly to 100 meters and activated the camera via UHF remote, or used an automatic switch that was placed within the cat’s home-range and activated the camera when it came within 100 meters of the station. Cats were re-caught 7−30 days later using the same methods to retrieve cameras and download footage.

### Data analysis

Post release footage was scanned and behaviour was classified into one of seven states:

- Sleep: No sign of consciousness, only occasional shifts of posture
- Rest: No change of position, but signs of alertness and movement.
- Grooming: Licking fur or scratching
- Drinking
- Walking: steady walking of less than two steps per second
- Running: vigorous movement of more than two steps per second
- Hunting: Any behaviour in which a cat appeared to have detected prey, usually characterised by stalking followed by a pounce or prey retrieval; some other prey hunting behaviour was also witnessed (e.g. cat walking up to birds eggs and eating them).

Brief switches in behaviour (states that lasted for less than 10 seconds, before reverting to the previous behaviour) were ignored.

We calculated the percentages of time spent in each behaviour class. The first 30 minutes post release was discarded, as cats were generally running away. To test for other distortions of behaviour due to capture and release, we compared the proportion of each behaviour from footage acquired less than eight hours to that obtained from cameras remotely activated more than two days post release using a MANOVA [24]. We calculated hunting rates and success rates from the time of each deployment, and differentiated between day and night footage. Animals killed but not consumed were considered as surplus killing. All hunting events, whether successful or unsuccessful, were examined in further detail. We recorded the behaviour state (as above) of the cat immediately before the prey was detected, whether the cat then initiated a stalk or pounce, the diel period (day, night or one hour either side of sunrise or sunset), the species of prey where possible, success of hunt (whether the prey was killed), details of prey consumption, and microhabitat. Microhabitat was recorded for the area of the stalk, and approximately 50 cm around the point of predation event, based on footage from the camera. For both, microhabitat was divided into three categories; *open* (little/no grass above 10 cm, no rocks with crevices or caves), *grass tussocks* (any grass above 10 cm) and *rock area* (rocks with noticeable crevices or caves). Only microhabitat could be considered, as opposed to macrohabitat, as we could not determine landscape position from camera footage. A demonstration of how microhabitat was assessed is provided in Supporting information S1 Video.

To examine the influence of vegetation structure on hunting success, we created multiple generalised linear models with binomial distributions. Model variables included diel period, stalk microhabitat, and prey location microhabitat. The one instance where food was acquired but no stalk or pounce was initiated (i.e. eating birds eggs) was not considered in this analysis. We did not include identities of individual cats as random error terms, as variation in hunting success between individual cats contributed very little to the overall variation (< 4%). Models were created with each variable and all combinations of variables, and we determined which variables best predicted hunting success within an information theory framework, calculating model weights and the relative importance of variables on the entire model set included in the analysis.

### Ethical statement

All field methods used in this study were approved by the Western Australian Department of Parks and Wildlife Animal Ethics Committee (2010/35 and 2013/40), with a Regulation 17 licence to research animals (SF009379). Field research was conducted with permission on Mornington Wildlife Sanctuary, owned and managed by the Australian Wildlife Conservancy, ph; +61 8 9191 7014; and Glenroy Station ph: +61 8 9191 4703. We confirm that the field studies did not involve endangered or protected species.

## Results

**Fig 2:**
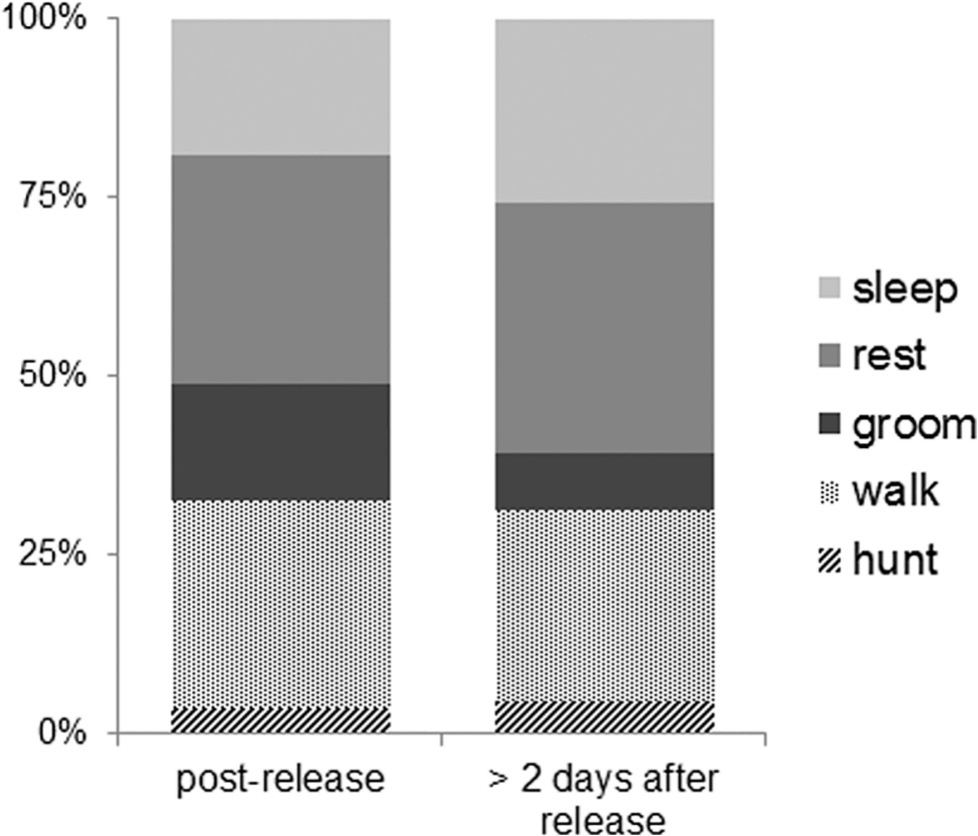
Activity budgets of feral cats during footage obtained within eight hours of capture and release (left column, 61 hours) and footage obtained two or more days post release (right, 28 hours).

From 23 deployments of video camera-collars on 13 individual feral cats between 2012 and 2014 (see Table 1), we acquired 98 hours of video footage. As we excluded the first 30 minutes after release of a cat, 89 hours of footage were analysed. There were no significant differences between behaviours less than 8 hours and more than 24 hours post release (approx. F_6, 16_ = 1.528, P = 0.232, Fig 2). The length of useable footage per deployment was on average 3:53 hours, and ranged from 1:37 to 8:18 hours. Cats were active (either the walking, running or hunting behaviours) for at least 10% of all deployments, with an average of 35% (SE = 3.60). Hunting behaviours were witnessed during 21 of the 23 deployments.

**Table 1:** Details of the 23 cat camera-collar deployments, including cat weight at start of footage, weight of collar compared to body weight, whether the start of footage was more than 24 hours post release (Delay), and length of useable footage for analysis.

Cat name	Sex	Cat weight (kg)	Collar weight (g)	Body weight %	Delay	Time	Useable footage (h:m)
Diddles	female	3.3	108	3.3	yes	night	4:53
Ruby	female	3.1	100	3.2	no	day	2:25
	female	2.9	100	3.4	no	night	2:33
	female	3.3	100	3.0	yes	day	2:26
Sophisticat	female	2.8	100	3.6	no	day	1:38
	female	3.3	100	3.0	no	day	2:19
Bazza	male	4.5	140	3.1	no	night	7:16
	male	4.5	100	2.2	yes	day	2:38
Blackbeard	male	4.4	128	2.9	no	night	3:26
Captain2	male	4.5	100	2.2	no	day	1:37
	male	4.7	128	2.7	no	night	3:17
	male	4.5	128	2.8	no	night	3:35
Darcy	male	5.0	100	2.0	no	day	2:31
Eyegore	male	4.3	100	2.3	no	day	2:17
Ginja ninja	male	5.0	128	2.6	yes	day	4:33
Jaws	male	4.4	120	2.7	no	night	5:54
Lee	male	5.7	120	2.1	no	day	4:17
	male	5.3	140	2.6	no	night	7:23
	male	5.0	128	2.6	yes	night	5:38
Mike	male	4.5	100	2.2	no	night	3:41
	male	4.8	140	2.9	no	night	8:18
	male	4.6	128	2.8	yes	night	4:13
Pork noodle	male	3.2	100	3.1	no	night	2:32

### Hunting rates and prey

From the 89 hours of footage considered, we recorded 101 hunting events, 32 of which were successful (see Table 2), for a rate of 0.3 (SE = 0.09) kills.hour^-1^, equivalent to 7.2 animals killed per 24 hours per cat. We recorded an average of 0.43 kills.hour^-1^ at night (SE = 0.05) and 0.15 during the day (SE = 0.11). Nine prey taxa were identified. Frogs comprised 44% of killed prey (see Table 2), but were eaten in only 50% of kills. Prey were not consumed in 28% of kills. S1 Video presents footage of many of these hunts.

**Table 2:** Prey species observed predated upon by feral cats, and details of body parts eaten.

Common name	Species	*N*	Details of consumption
Locust	unknown	1	eaten whole
Giant frog	*Cyclorana australis*	3	stomach and legs eaten (1), uneaten (2)
Rocket frog	*Litoria nasuta*	2	eaten whole (1), head eaten (1)
Frog	various *spp*.	7	eaten whole (1), head eaten (2), uneaten (4)
Green tree frog	*Litoria caerulea*	2	stomach and legs eaten (1), uneaten (1)
Gilbert's dragon	*Lothognathus gilberti*	2	eaten whole (2)
Gecko	unknown	1	eaten whole
Western brown snake	*Pseudonaja nuchalis*	1	head chewed off, body half eaten
Shovel-nose snake	*Brachyurophis roperi*	1	eaten whole
Brown quail	*Coturnix ypsilophora*	2	eaten whole
Birds eggs	unknown	1	Only yolk eaten
Common rock-rat	*Zyzomys argurus*	1	eaten whole
Western chestnut mouse	*Pseudomys nanus*	5	eaten whole (4), uneaten (1)
Unknown		3	
**Total**		**32**	

**Fig 3:**
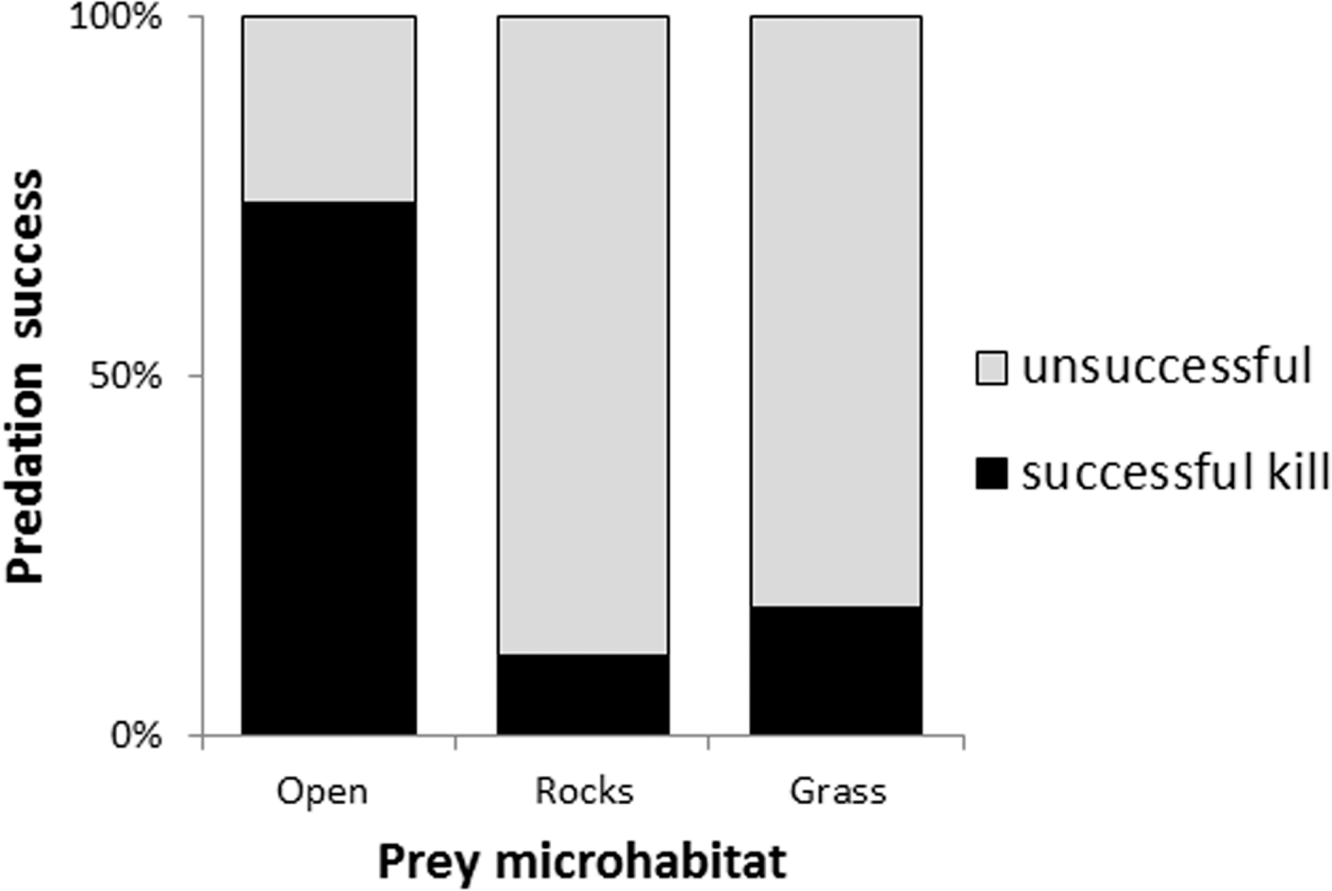
Portion of successful and unsuccessful predation events when prey was located either in the open (no grass cover over 10 cm), rocks, or grass tussocks.

Once cats had initiated a hunt by either stalking and/or pouncing, they were successful in 30% of cases. Both of the two top-ranked models describing success rate (delta AIC < 2, see Table 3) contained the microhabitat variable (relative importance = 0.999). Variables of stalk microhabitat and interactions with deil period did not feature in the top candidate models. Hunts were 4.1 times more likely to succeed if the prey was located in open habitat than a closed habitat (see Table 3). This translates to a 70% chance of a kill in an open area, and 17% chance in an area with dense grass or complex rocks (see Fig 3). The second ranked model also suggested that hunts were more successful at night (relative importance = 0.23).

**Table 3:** Details of the top candidate models, null model and global model. Other nine models with delta > 2 not shown.

Model	Intercept	Open area	Night	df	logLik	AICc	Delta AIC	weight
1	-1.6	2.5		2	-49.2	102.5	0	0.44
2	-1.8	2.5	0.3	3	-49.1	104.4	1.9	0.17
Global	0.1	14.6	1.6	6	-48.9	110.6	8.1	0.01
Null	-0.8			1	-62.0	128.2	25.6	0
Relative importance of variable		0.99	0.35					

## Discussion

Once a prey animal had been detected, the success rate of hunts by feral cats was strongly affected by microhabitat. Feral cats were over four times more likely to make a successful kill when the prey was located in an open microhabitat, than if it was in complex vegetation or rocks. Grass tussocks and rocks are thus highly valuable in providing refuge for prey, and when present they prevented the success of 82% of hunting events. Presence of cover during the stalk did not improve model fitness, and whilst it still might have had an influence that wasn’t detected, it is clear that the principle variable was the importance of prey microhabitat. GPS tracking in the same area revealed strong preferences by cats for open habitats, such as fire scars and heavily grazed areas [21]. Our new results suggest that the reason for this preference is the higher hunting success that cats would achieve in these habitats. This result is consistent with studies describing mortality in small mammals in northern Australia, where survival is consistently lower in areas with open microhabitats versus areas with dense grass or rocks [25–28].

The weight and bulk of our camera-collars did not appear to cause distress in cats, in that there was no footage obtained of cats in obvious discomfort or attempting to remove collars. The collars are unlikely to have reduced hunting success, as the 30% reported here is very similar to hunting successes recorded in other observational studies of feral cat hunting [29–31]. Although one study suggests the weight of collars greater than 2% could reduce cat home range by 15% [32], this study used domestic cats with home ranges around one ha. The cats used in this study all carried separate GPS collars of similar weight prior to use in this study [33], and there was no evidence of contracting home-ranges, despite home ranges being much larger (average 800 ha) and presumably much harder to move around. The most frequent prey type taken by cats in our study was frogs. Other cat diet studies, based on stomach analyses, have not reported frogs as a major prey item [13,14,34,35]. We found that frogs were rarely eaten whole and hard parts were often discarded. Analysis of gut contents would therefore underestimate predation on frogs. Snakes, ground-dwelling birds and small mammals were the other main prey taxa, similar to other studies [35]. Interestingly, feral cats can possibly discriminate among snake species. In a case where the highly venomous western brown snake *Pseudonaja nuchalis* was eaten, the cat spent almost 10 minutes chewing off the head, which it then discarded, perhaps to avoid ingestion of venom. However, a different individual cat quickly ate a shovel-nose snake *Brachyurophis roperi* whole, which is not sufficiently venomous to be dangerous to cats.

A large proportion of animals killed were not eaten (28%). This tendency towards ‘surplus killing’ means that kill rates may increase in circumstances where more prey is available to cats; if prey is more abundant, and/or when prey are unable to avoid detection. Such circumstances may include areas affected by heavy stock grazing and intense fires, where all ground cover has been removed and prey are exposed [36]. However, most unconsumed prey in this study was frogs. Therefore, it is possible that the surplus killing recorded in this study was related to toxicity in frogs [37]. Further video evidence will be required to assess the rate of surplus killing of other prey.

Feral cats succeeded in making a kill in 30% of recorded hunts, and killed over seven animals per day. Estimates of kill rates by free-ranging domestic cats that had supplementary feeding are 20 times lower than recorded here, at 0.34 prey items per night [13], indicating that feral cats have a far greater impact on prey populations than domestic cats [15,16,38]. Kill rates are also much lower for large carnivores, with lynx *Lynx lynx* as low as 0.1 to 0.3 kills per day per capita [39] and lions *Panthera leo* 0.4 kills per day per capita [40]. These larger predators hunt prey that are larger relative to their body size than for feral cats, capturing each prey item is a more intensive activity, and a single large prey item can provide enough food for many days.

We cannot use these data to assess whether cat predation is detrimental to prey populations; our dataset is too small and there are too many other unknown variables we could not measure. Hunting success would differ among prey types (e.g. large frogs might be easier to hunt than quail), but we could not quantify this, as we do not know the prey species for most unsuccessful hunts. Also, we could not link hunting rates to prey abundances, as the nocturnal video footage could not reveal the broad habitat type (e.g. spinifex grassland, riparian forest), and prey abundances vary markedly between these [23]. Although we cannot comment as to whether the seven animals killed per night would constitute a threat to prey populations, we do demonstrate the potential for a four-fold increase in predatory impacts if ground cover is altered in ways that provide more open microhabitats (like after an intense fire).

Many Australian mammal species have declined across the continent and persist only in small areas of the mainland [41], usually in structurally complex habitats. For example, the Gilbert’s Potoroo *Potorous gilbertii* [42] which was once widespread, retracted to a single location of long-unburnt structurally complex coastal heath. Similarly, native mammal extinctions have been more prevalent in regions with relatively more open habitats, such as deserts and grasslands [19,41,43,44]. By demonstrating the influence of habitat complexity on hunting success of feral cats, our data offer a potential mechanistic understanding as to why predation by feral animals is mitigated in some areas.

## Conclusions

The patterns observed in our study are likely to be applicable to other small felid predators, as most are opportunistic hunters that preferentially hunt prey much smaller than themselves [45]. This means that dense and complex microhabitats are likely to decrease predation rates, as prey could move and take refuge into spaces where the predator cannot follow.

That cover during the stalk did not have a significant or substantial influence on predation success suggests that cat predatory impacts could be greater in totally barren landscapes, compared to mixtures of open areas and dense grass. This compliments numerous studies in northern Australia on persistence of small mammal populations [25,27,28,46]. However, our research only supports this in context of if a cat detects prey. There are many other factors affecting overall predatory impacts that were not considered here. For example, cats are prey to larger predators themselves, such dingoes *Canis lupus dingo*. It is possible cats themselves are more vulnerable in open habitats as well. As such, they might only hunt in such habitats when safe to do so [47], or select for edge habitats where retreat is possible [21]. While further research will be required to determine whether barren landscapes are more detrimental to prey populations than mixtures of open areas and dense grass, this study at least suggests that any such patterns would not be driven by differences in cat hunting success.

Our research demonstrates a broad principle for reducing the impacts of cat predation, which is to increase the spatial and temporal cover of ground vegetation wherever possible, thus providing refuges for prey to hide. This might be achieved by supressing intense fires, reducing populations of feral herbivores, and restoration of dense grass or shrubs.

## Supporting Information

S1 Video For this supporting information, a short video was created, providing a brief overview of the footage and how we determined characteristics of hunting. (MP4)
